# Heritability and Associations among Grain Yield and Quality Traits in Quality Protein Maize (QPM) and Non-QPM Hybrids

**DOI:** 10.3390/plants11060713

**Published:** 2022-03-08

**Authors:** Isaac Kodzo Amegbor, Angeline van Biljon, Nemera Shargie, Amsal Tarekegne, Maryke T. Labuschagne

**Affiliations:** 1 Department of Plant Sciences, University of the Free State, Bloemfontein 9301, South Africa; avbiljon@ufs.ac.za; 2 Maize Improvement Programme, CSIR-Savanna Agricultural Research Institute, Tamale P.O. Box TL 52, Ghana; 3 ARC—Grain Crops Institute, Private Bag X1251, Potchefstroom 2520, South Africa; shargien@arc.agric.za; 4 Zambia Seed Company Limited (Zamseed), Lusaka P.O. Box 35441, Zambia

**Keywords:** path coefficient analysis, principal components, correlations, heritability

## Abstract

Maize (*Zea mays* L.) is the main staple cereal food crop cultivated in southern Africa. Interactions between grain yield and biochemical traits can be useful to plant breeders in making informed decisions on the traits to be considered in breeding programs for high grain yield and enhanced quality. The objectives of this study were to estimate the heritability of grain yield and its related traits, as well as quality traits, and determine the association between quality protein maize (QPM) with non-QPM crosses. Grain yield, and agronomic and quality trait data were obtained from 13 field trials in two countries, for two consecutive seasons. Significant genotypic and phenotypic correlations were recorded for grain yield with protein content (r_G_ = 0.38; r_P_ = 0.25), and tryptophan with oil content (r_G_ = 0.58; r_P_ = 0.25), and negative r_G_ and r_P_ correlations were found for protein with tryptophan content and grain yield with tryptophan content. Path analysis identified ear aspect, ears per plant, and starch as the major traits contributing to grain yield. It is recommended that ear aspect should be considered a key secondary trait in breeding for QPM hybrids. The negative association between grain yield and tryptophan, and between protein and tryptophan, will make it difficult to develop hybrids with high grain yield and high tryptophan content. Hence, it is recommended that gene pyramiding should be considered for these traits.

## 1. Introduction

Maize is one of the principal staple food crops grown and consumed in South Africa and Zimbabwe; however, most of the maize varieties under cultivation are deficient in essential amino acids. The crop has the potential of enhancing food security in the sub-region, as well as to combat malnutrition. The interactions between grain yield and biochemical traits can be useful to plant breeders in making informed decisions on traits to be considered in maize breeding programs to achieve high grain yield and essential quality traits.

Breeding for biofortified crops with enriched nutritional quality can help improve nutritional deficiencies. Non-QPM, which is generally cultivated, provides little or no nutritional benefits as food and feed for humans and other monogastric animals. Maize provides micronutrients, such as vitamin B complex and ß-carotene, and essential minerals, such as magnesium, zinc, phosphorus, and copper. However, the endosperm of non-QPM is deficient in two essential amino acids, lysine and tryptophan, despite the endosperm containing approximately 10% protein, 73% starch, and 4% oil [1]. The development, adoption, and cultivation of QPM with higher concentrations of tryptophan content can significantly reduce malnutrition and its related diseases [2]. Lysine and tryptophan have to be supplied through the diet to ensure adequate availability for the synthesis of proteins. Therefore, lysine and tryptophan are often considered as the most essential amino acids for the body, while the remaining amino acids are considered non-essential, since they can be synthesised through metabolism [3].

The heritability and association of traits are some of the important components considered in breeding for superior hybrids. Despite the importance of heterosis in maize production being reported, the inherent mechanisms and, most importantly, the physiological and biochemical mechanisms of these phenomena, are yet to be fully elucidated [4]. In most breeding programs, increased yield is the primary objective. As a result of the interactions between genotype and the environment, the full expression of grain yield and other traits, which are quantitatively inherited, fluctuate under varying environmental conditions [5]. For the selection of grain yield and quality traits to be efficient, it is important to consider traits that contribute to yield and quality. The polygenic nature of grain yield often leads to variability across different environmental conditions [6,7]. Subsequently, grain yield and quality trait enhancement in maize can be realised via the utilisation of the association between grain yield, quality traits, and their associated characters [8,9]. Such correlations have been exploited in several studies for the indirect selection of yield and quality traits [8,9,10].

In relation to breeding for QPM hybrids with enhanced quality traits, it is important that protein quality has a positive relationship with other kernel quality characteristics, to ensure the success of breeding for quality traits. The study of the correlations between the quality traits of maize, such as endosperm hardness, protein quality, starch, and other traits related to endosperm texture, is important for kernel modification in breeding programs, by crossing genotypes with hard kernels with genotypes with higher amounts of amino acids but with softer kernels [11]. Several reports indicated that, as the amount of protein and tryptophan increased, there is also a decrease in endosperm modification [12]. In addition, Sharma et al. [13] noted that traits, such as number of kernels per cob, tryptophan and lysine contents, and grain yield per plant, showed high values for heritability, genetic advance, and genetic correlations. Jilo [11] reported a negative relationship between grain yield and protein quality, and grain yield and kernel modification; hence, there is a need to conduct chemical analysis on the maize endosperm for tryptophan before classifying a genotype as QPM [14]. Pixley and Bjarnason [15] and Aliu et al. [14] reported insignificant genotypic correlation between endosperm texture, protein content, and grain yield.

Information on the correlations between traits is crucial in maize breeding to aid the identification of superior genotypes with higher grain yield through indirect selection, achieved via the selection of secondary traits [16]. However, it is important to note that correlations among traits are not adequate to describe the significance of each character contributing to grain yield [17]. These inadequacies often lead to observed dissimilarities that are due to more than one indirect cause [18]. As a result, it is important to conduct in-depth studies on trait associations to fully understand the contribution of each trait, and then rank their importance for targeted selection. One of the ways of achieving this is to use path coefficient analysis to assess the direct and indirect relationships among traits [9,19]. The objectives of this study were to (i) estimate the heritability of grain yield, its related characters, and quality traits, and (ii) determine the association among grain yield, and agronomic and quality traits in QPM with non-QPM crosses.

## 2. Results

### 2.1. Variance Components and Heritability for Grain Yield, and Agronomic and Quality Traits

Estimates of tester variance were higher than line variance for anthesis-silking interval, plant and ear height, grain yield, and stalk lodging; alternatively, line variance was higher for ear rot, husk cover, days-to-anthesis, and root lodging, although values were generally low (Table 1). Genetic variance was larger than environmental variance for grain yield, plant and ear height, ears per plant, ear aspect, ear rot, and days-to-pollen shed. The environmental variance was higher for anthesis-silking interval, husk cover, and root and stalk lodgings. Broad-sense heritability (H^2^) estimates were generally higher than 80% (Table 1).

For the quality traits, the estimates of tester variance were high for tryptophan, protein, and fibre, while line variance was also high for moisture, oil content, and starch. Genetic variances were relatively low for tryptophan, moisture, and oil content, but relatively high for protein and starch content. The genotypic variances were higher than the environmental variance for all the traits analysed. Furthermore, additive variances were larger than dominance variances for tryptophan, oil content, moisture, fibre, starch, and protein. Broad-sense heritability (H^2^) estimates were high (above 90%) for all the quality traits (Table 1).

### 2.2. Principal Component Analysis

The first four principal components (PCs) explained 96.4% of the variation of the agronomic traits measured (Table 2). The first four PCs were significant, with PC1 accounting for 80.10% of the variation. The most important traits in PC1 were plant and ear height. The second, third, and fourth PCs explained 6.90, 5.30, and 4.10% of the variation, respectively. The most important traits in these PCs were plant height in PC2, and ear rot and ear height in PC3. Root and stalk lodging were the important traits in PC4.

**Figure 1:**
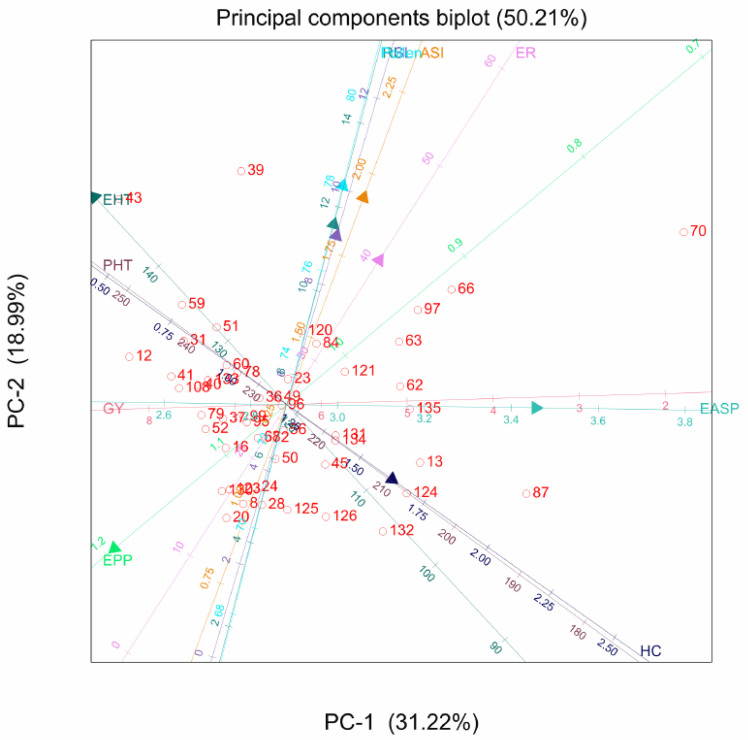
Principal component analysis biplot of genotype-by-grain yield and other agronomic traits of 135 maize hybrids: GY, grain yield; EPP, ears per plant; PHT, plant height; EHT, ear height; EASP, ear aspect; HC, husk cover; SL, stalk lodging; RL, root lodging; ASI, anthesis-silking interval.

The PCA biplot showed that the hybrids varied for the agronomic traits. Genotypes 43 and 109 recorded high values for ear and plant height, respectively. Genotypes 123 and 69 were the most prolific hybrids, while genotypes 79 and 37 had a higher grain yield. The angle between ears per plant and grain yield is less than 60°, suggesting a strong correlation between the two traits. Similarly, ear and plant heights are strongly correlated based on the angle between these traits. Grain yield and ear aspect are directly opposite on the graph, indicating a negative correlation between them (Figure 1).

The PCA of the quality traits showed that the first three PCs accounted for 99.70% of the total variation (Table 3). However, only the first three PCs were significant, of which PC1 accounted for 63.70% of the variation. The most important traits on this axis were grain moisture and starch content. The second PC explained 20.50% of the variation, with protein, oil content, and starch as the most important traits on this axis; the third PC explained 15% of the variation, the important traits being protein, oil, and starch content.

**Figure 2:**
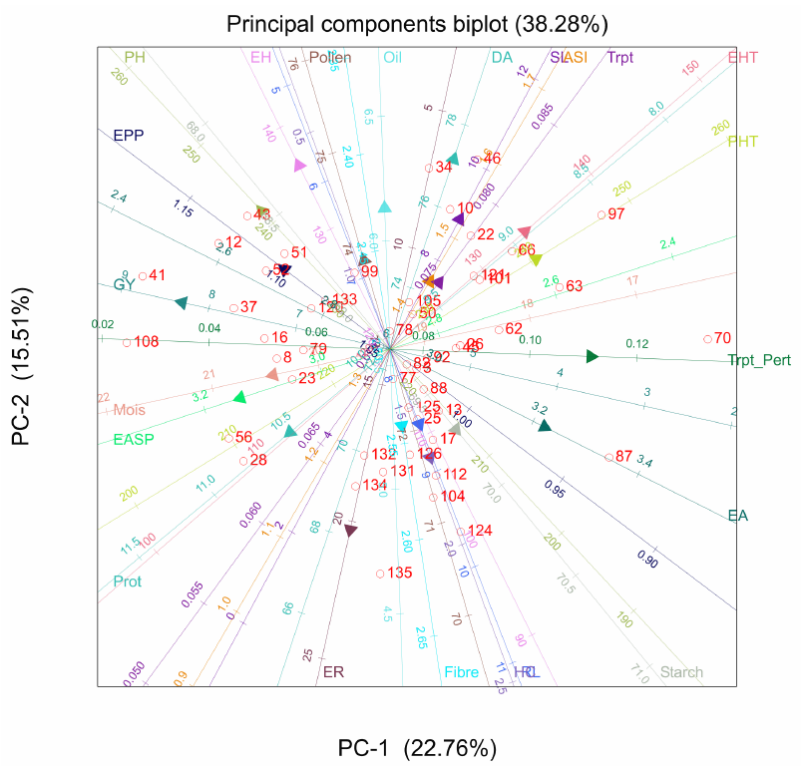
Principal component analysis biplot of genotype by quality traits of 135 QPM and non-QPM hybrids evaluated across six locations: Trypt_Pert, tryptophan; Prot, protein.

The PCA biplot for quality traits indicated significant variations among the hybrids. A strong positive correlation was detected between fibre and tryptophan, with hybrid 84 having the highest values for these traits. Hybrids 43 and 31 were the best genotypes for oil content. Protein and tryptophan were not positively correlated, and similar observations were made between protein and oil content (Figure 2).

When the agronomic traits were combined with the quality traits, six significant PCs were identified, and these explained 98.40% of the variation for the measured traits (Table 4). The first PC explained 78.80% of the variation, and the traits located on this PC were ear and plant height. PC2 accounted for 6.80% of the variation, and the traits contributing to PC2 were plant and ear height, and ear rot. The third and fourth PCs accounted for only 5.3 and 4.10% of variation, respectively (Table 4).

**Figure 3:**
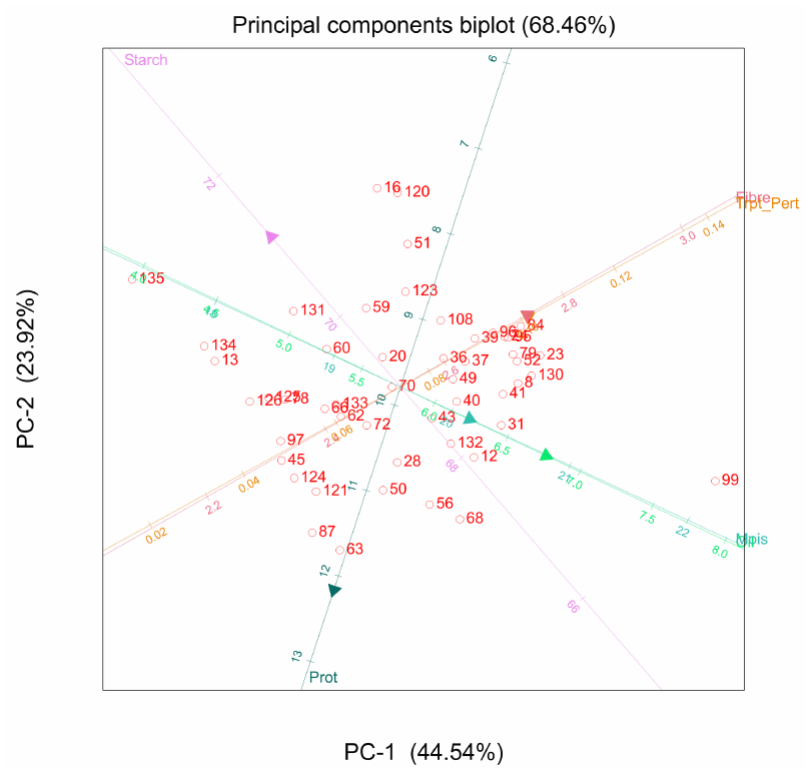
Combined principal component analysis biplot of 135 genotypes for grain yield, agronomic and quality traits: GY, grain yield; EPP, ears per plant; PH, plant height; EH, ear height; EASP, ear aspect; HC, husk cover; SL, stalk lodging; RL, root lodging; ASI, anthesis-silking interval; Trypt_Pert, tryptophan; Prot, protein.

When all agronomic and quality traits were combined, most of the agronomic traits clustered together, as did the quality traits. Positive correlations were evident for grain yield, plant height, ear height, and ears per plant, with the angles between them measuring less than 90°. However, oil content was highly correlated with anthesis-silking interval, with an angle less than 45° between them. Tryptophan content also showed a highly positive correlation with stalk lodging (Figure 3).

### 2.3. Genotypic and Phenotypic Correlation between Grain Yield and Other Agronomic Traits

For genotypic correlation, grain yield was significantly and positively correlated with plant height, ear height, and ears per plant, while it was negatively correlated with husk cover, stalk lodging, and ear aspect (Table 5). Ears per plant was also positively and significantly correlated with plant and ear height, but negatively correlated with anthesis-silking interval, root lodging, and husk cover. Ear aspect was significantly and positively correlated with husk cover, and negatively correlated with plant and ear height, stalk lodging, and husk cover. Similar to the genotypic correlation, grain yield was significantly and positively phenotypically correlated with plant height, ear height, and ears per plant. Grain yield showed a negative and significant correlation with ear aspect (r_P_ = −0.61), and also showed negative correlations with stalk lodging and husk cover (Table 5).

Tryptophan content showed a significant positive genotypic association with moisture, oil, and fibre content, but a strong negative correlation with protein and starch content. Protein had a significant and negative relationship with fibre and starch, but correlated poorly with oil. Oil content showed a strong positive correlation with fibre and a highly negative correlation with starch (r_G_ = −0.78) (Table 5). Tryptophan exhibited a positive phenotypic correlation with oil content and fibre, while a significant negative correlation was recorded between tryptophan and protein (r_P_ = −0.559) and tryptophan and starch (r_P_ = −0.299). Protein showed negative and significant relationships with all the quality traits, except for oil content (Table 5). Oil content showed a negative correlation with starch but positively correlated with fibre.

Grain yield had positive genotypic association with moisture and protein content; however, regarding phenotypic correlation, grain yield correlated positively with protein, while negative association was observed between grain yield and starch (Table 5). Ear aspect correlated significantly and positively with tryptophan and fibre content, but correlated negatively with protein content. Ear height was significantly and negatively correlated with tryptophan and fibre content (Table 5). Grain yield showed negative and significant phenotypic correlation with tryptophan, moisture, and protein content. Ear aspect showed a weak positive (r_P_ = 0.19) correlation with tryptophan (Table 5).

### 2.4. Path Coefficient Analysis for Grain Yield and Agronomic Traits

**Figure 4:**
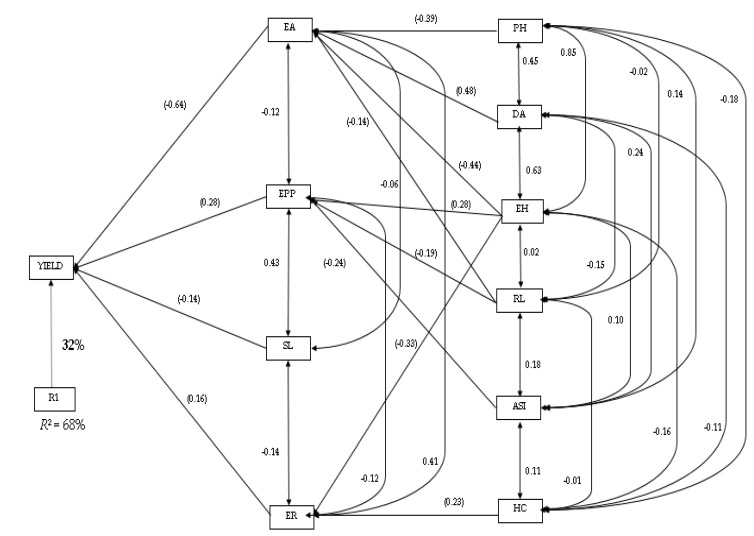
Path analysis showing the relationship of grain yield and agronomic traits of maize hybrids: yield, grain yield; ER, ear rot; EPP, ear per plant, EA, ear aspect, HC, husk cover, ASI, anthesis-silking interval; EH, ear height; PH, plant height; DA, days-to-anthesis; RL, root lodging; SL, stalk lodging. Values in parentheses are correlation coefficients, and other values are direct path coefficients. R1 represents the residual effects.

The path coefficient analysis, using the stepwise regression model for the grain yield and agronomic traits measured, identified ear aspect, ears per plant, stalk lodging, and ear rot as the traits that contributed to grain yield most directly, and accounted for 68% of the variation in grain yield (Figure 4). Ear aspect recorded the highest direct effect (−0.64) on grain yield, while ear rot had the least direct effect on grain yield (Figure 4). Plant and ear height, anthesis-silking interval, husk cover, days-to-anthesis, and stalk lodging were ranked as the second order of traits. With the exception of husk cover and anthesis-silking interval, the rest of the second order traits contributed to grain yield indirectly via ear aspect. Anthesis-silking interval and root lodging contributed to grain yield through ears per plant, while husk cover contributed to grain yield through ear rot. However, none of the second order traits contributed to stalk lodging to grain yield.

**Figure 5:**
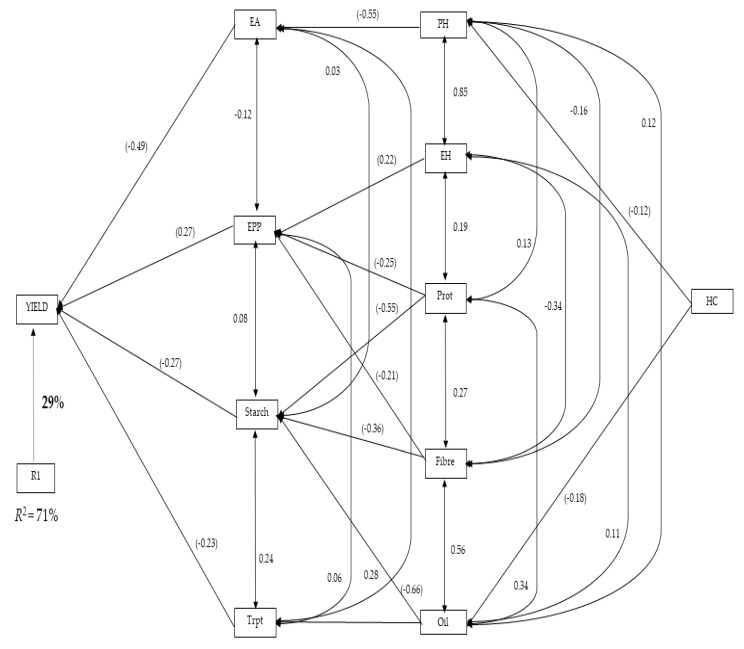
Path analysis showing the relationship of grain yield, agronomic and quality traits of 135 QPM and non-QPM maize hybrids: yield, grain yield; ER, ear rot; EPP, ear per plant, EA, ear aspect, HC, husk cover, ASI, anthesis-silking interval; EH, ear height; PH, plant height; DA, days-to-anthesis; Trypt, tryptophan; Prot, protein. Values in parentheses are correlation coefficients, and other values are direct path coefficients. R1 represents the residual effects.

The path analysis (Figure 5) shows the stepwise regression model for grain yield, with secondary traits related to grain yield and quality traits. The first order traits contributing directly to grain yield were ear aspect, ear per plant, starch, and tryptophan; these contributed 71% of the variation of grain yield (Figure 5). Ear aspect contributed the highest direct effect (−0.49) on grain yield, while tryptophan recorded the lowest effect (−0.23). Plant and ear heights, protein, fibre, and oil content were the other traits contributing to grain yield indirectly. Oil content, fibre, and protein contributed to grain yield through starch; alternatively, ear height, protein, and fibre contributed to grain yield indirectly through ears per plant. Only plant height contributed to grain yield through ear aspect, while oil content also contributed to grain yield via tryptophan for the second order traits. Husk cover was identified as the only third order trait contributing to grain yield through plant height and ear aspect, and also through oil content and tryptophan.

## 3. Discussion

### 3.1. Heritability and Variance Components for Agronomic and Quality Traits

Substantial phenotypic and genotypic variations were evident among the QPM and non-QPM hybrids. High broad-sense heritability values suggest that selection is possible for superior inbred genotypes, confirming earlier findings [20,21]. However, the heritability estimates in this study were higher than the previous estimates. Dutta et al. [22] and Mastrodomenico et al. [23] indicated that phenotypic variance and heritability are indicators of direct selection. For the genotypes studied, the genotypic variances (σ^2^g) were higher than the environmental variances (σ^2^e) for most of the traits measured, excluding husk cover, root, and stalk lodging. This is consistent with previous reports [20,21], suggesting that the recently developed QPM inbred lines of the CIMMYT are suitable for developing superior hybrids for grain yield and other phenotypic traits.

The high broad-sense heritability (H^2^) values recorded for the various quality traits in the present study indicate that the identification and selection of inbred lines with increased tryptophan, protein, starch, and oil content, and low fibre content, is possible to reduce malnutrition. The higher values further indicate the genetic variability in the material studied, hence some of the genotypes can be selected for synthetic cultivar or population development, from which other superior quality traits, such as tryptophan, protein, and oil content could be developed.

Selection based only on traits with high H^2^ is simple due to the limited influence of the environment on these genotypes [24]. However, H^2^ alone is not adequate to enable selection for promising individuals; thus, it is important to include other genetic components [10]. This study showed that σ^2^g was higher than σ^2^e for protein, tryptophan, oil, starch, and fibre content. This corroborates previous findings [25,26], that many quality traits are controlled by additive genes.

### 3.2. Principal Component Analysis for Agronomic and Quality Traits

For the PC biplot of the agronomic traits, PC1 and PC2 accounted for 51.25% of the variation in this dataset, with a strong association between grain yield and ears per plant, and plant and ear height; indicating that prolificacy is a major contributor to yield, coupled with plant and ear height. For the quality traits, a large contribution from ASI and protein content were observed in PC1 and PC2. This result is partly consistent with a previous report [27], in which major amino acids, such as leucine and lysine, were located on the first PC. The strong association between tryptophan and fibre, as evident in the PC biplot, suggested that increased tryptophan will lead to higher fibre content. Similarly, the angle between tryptophan and oil content is less than 90°, suggesting a strong relationship between the traits. However, tryptophan and protein cannot be selected simultaneously, due to their negative relationship [28].

For the combined agronomic and quality traits, the characters used different pathways. While the agronomic traits were located on the first four PCs, the quality traits used were mainly located on the fifth and sixth PCs.

### 3.3. Correlation Coefficients and Path Analysis of Grain Yield, Agronomic and Quality Traits

Genetic and phenotypic correlations showed similar trends for the traits analysed, hence this discussion applies to both phenotypic and genotypic correlations. The substantial negative relationship between grain yield and anthesis-silking interval, and grain yield and ear aspect, indicates that these traits are inversely related; therefore, they could be considered as essential traits in breeding for QPM genotypes aiming at high grain yield with reduced days-to-maturity. This confirms previous reports [29,30]. The positive and significant correlation between grain yield and number of ears per plant, grain yield and plant height, and grain yield and ear height, indicates that these traits can be used for selecting high-yielding QPM genotypes, corroborating the findings of other studies [29,31,32]. Husk cover has significant implications for ear rot at genotypic and phenotypic levels, signifying that closed cob tips should be considered as an important secondary trait to prevent the exposure of kernels from adverse environmental conditions, and also from insect attack, which consequently affects grain yield and quality. Grain yield was negatively associated with tryptophan and starch content, indicating that these traits cannot be selected together; this in turn implies pleiotropy, thereby, as one increases, the other decreases. The strong positive relationship between grain yield and protein indicates that these traits can be selected together. The strong negative correlation between protein and tryptophan content, and starch and oil content, indicates that the simultaneous improvement of these traits will be difficult, as the increase in one will decrease the other, corroborating the findings of Pixley and Bjarnason [33]. The correlation between grain yield and protein content contradicts the results of a previous study [34] that recorded a strong negative association between grain yield and protein, although it agrees with another study [35], in which the authors also reported a positive and significant association between grain yield and protein content. The repeated selection for high protein content and yield has probably reduced the negative correlation between these two traits.

Due to the polygenic nature of grain yield, selection based only on correlation may not be efficient for selecting superior genotypes; therefore, it is crucial that we access other pathways through which grain yield is inherited. Path coefficient analysis assists plant breeders in detecting favourable traits that aid selection to enhance grain yield [36,37]. The path coefficient analysis was conducted for grain yield against other agronomic traits, and to determine how the combination between agronomic and quality traits are related to grain yield. The study identified ear aspect as a major contributor to grain yield, also serving as a channel through which several secondary traits contribute to grain yield indirectly. The identification of ear aspect in this study is consistent with the results of previous studies [38], indicating that ear aspect is a major trait contributing to grain yield. Ears per plant were also identified as a direct and indirect means through which ear height, and protein and fibre content contributed to grain yield. Additionally, starch and husk cover contributed indirectly to grain yield through tryptophan. Path analysis has also been utilised by several authors for other crops, such as tomato [39], groundnut [40], and rice [41].

## 4. Materials and Methods

### 4.1. Field Trials

The genetic materials used in this study were QPM and non-QPM hybrids developed by the International Maize and Wheat Improvement Centre (CIMMYT). A total of 130 hybrids were developed by crossing 33 inbred lines (23 QPM and 10 non-QPM) with four testers (two QPM and two non-QPM), including five commercial check hybrids (three QPM and two non-QPM). The hybrids were selected based on their yield potential and quality traits. Field experiments were conducted during the 2017/2018 and 2018/2019 cropping seasons at 13 locations in South Africa and Zimbabwe (Supplementary Table S1). The soil type at Harare is classified as Chromic Luvisol with a clay content of 40% and organic matter content with a noticeable rough structure [42]. The soil at Bindura is classified as Chromic Luvisol with heavy red clay soils of up to 40% clay content and rich in organic matter [42]. The soil type at Rattray Arnold is Harare 5G2 series. The soil at Potchefstroom consists of brownish sandy clay loam. The soil type at Cedara is sandy clay soil with adequate drainage. The weather data recorded across the sites and soil parameters analysed are presented in Supplementary Table S1. The trials were laid out in a 5 · 27 alpha lattice design with two replications. Fertilisers were applied at the recommended rate of 250 kg ha^−1^ N, 83 kg ha^−1^ P, and 111 kg ha^−1^ K. Basal fertiliser application was conducted in the form of NPK, and additional N application was conducted four weeks after emergence. In addition to grain yield, traits measured from the field experiments were plant and ear height, days-to-anthesis, husk cover, plant aspect, ear rot, and number of ears per plant. From the field experiments, two plants per plot were self-pollinated, which were used for laboratory analysis of protein, tryptophan, and starch. Since the study was conducted to determine the relationship between grain yield, and agronomic and quality traits, the hybrids were not partitioned into QPM and non-QPM for the analysis.

### 4.2. Determination of Tryptophan and Starch

For tryptophan, 50 kernels of uniform size per sample were milled into a fine flour using a Fritsch analysis grinder (LABOTEC, Johannesburg, South Africa). Samples were extracted and prepared [43]. A Jenway Spectrophotometer Model 7315 (Cole-Parmer, UK) was used for the optical density reading for tryptophan content. Total starch was determined by the polarimetric method [44].

### 4.3. Determination of Protein, Oil, Moisture, and Fibre

A total of 500 g of the self-pollinated seeds for each sample were used for protein, oil, moisture, and fibre content determination with near-infrared transmission spectroscopy (NIR) using a Perten Grain Analyzer (Model DA 7250, Perten, Instruments AB, Sweden). Prior to the use of the NIR, the equipment was calibrated by Agri-Envrion Solutions (www.aelab.co.za) using wet chemistry results of 50 samples. The correlation between the wet chemistry and the NIR values was more than 90%, indicating that the NIR readings reliable. The percentage of oil, protein, moisture, and fibre contents were expressed on a dry matter percentage weight basis (%wt).

### 4.4. Heritability Estimates

Broad-sense heritability (H^2^) of each trait across environments was estimated as follows:

H^2^_=_ σ^2^_g_ / σ^2^_p_ (1)

where σ^2^_g_ = genotypic variance, and σ^2^_p_ = phenotypic variance. The σ^2^_p_ was computed as follows:

σ^2^_p_ = σ^2^_g_ + σ^2^_g_/l + σ^2^_gt_/t + σ^2^_glt_/lt + σ^2^_e_/rlt (2)

where σ^2^_gl_ = genotype × location interaction variance, σ^2^_gt_ = genotype × treatment interaction variance, σ^2^ _glt_ = genotype × location × treatment interaction variance, σ^2^_e_ = environmental variance, r = no. of replications, l = no. of locations, and t = no. of treatments. 

Narrow-sense heritability (h^2^) was computed as follows:

h^2^= σ^2^_a_ / σ^2^_p_ (3)

where σ^2^_a_ = additive genetic variance.

### 4.5. Estimation of Variance Components

Additive and dominance variances were estimated from mean square values for testers, lines, and line x testers [45], as follows:

(4) $$\mathrm{Cov}\;\left(H.S\right)\mathrm{testers}=\left(\frac{\mathrm{MS}\;\mathrm{testers}-\mathrm{MS}\;\mathrm{line}\;x\;\mathrm{tester}}{\mathrm{rl}}\right)$$

(5) $$\mathrm{Cov}\;\left(H.S\right)\mathrm{lines}=\left(\frac{\mathrm{MS}\;\mathrm{lines}-\mathrm{MS}\;\mathrm{line}\;x\;\mathrm{tester}}{\mathrm{rt}}\right)$$

(6) $$\delta_{A}^{2}\left(\mathrm{testers}\right)=4\;\mathrm{Cov}\;\left(H.S\right)\mathrm{testers}$$

(7) $$\delta_{A}^{2}\left(\mathrm{lines}\right)=4\;\mathrm{Cov}\;\left(H.S\right)\mathrm{lines}$$

(8) $$\mathrm{Cov}\;\left(H.S\right)\;\mathrm{average}=\frac{\mathrm{Ml}+\mathrm{Mt}-2\mathrm{Mlxt}}{r\left(l+t\right)}$$

where Cov = covariance, MS = mean square, H.S = half sib families, r = no. of replications, l = no. of locations, t = no. of treatments, and σ^2^_a_ = additive genetic variance.

### 4.6. Principal Component Analysis

GenStat 20th edition statistical software [46] was used for the principal component analysis (PCA) to obtain the eigenvalues and PC biplots.

### 4.7. Genetic and Phenotypic Correlation Estimations

The raw data obtained for both the agronomic and quality traits were used to estimate genetic (r_G_) and phenotypic (r_P_) correlations using META-R (Multi Environment Trial Analysis with R for Windows) version 6.04 [47], using the following procedures:

Genotypic correlation (r_G_) = C_AXY_/(V_AX_V_AY_)^1/2^ (9)

where C_AXY_ = additive covariance between characteristic X and Y, V_AX_ = additive variance of characteristic X, and V_AY_ = additive variance of characteristic Y.

Phenotypic correlation (r_P_) = Cov_XY_/(σ^2^_X_σ^2^_Y_) (10)

where Cov_XY_ = phenotypic covariance between characteristic X and Y, σ^2^_X_ = phenotypic variance of characteristic X, and σ^2^_Y_ = phenotypic variance of characteristic Y.

### 4.8. Regression and Path Coefficient Analyses

A stepwise multiple regression analysis was performed using the data from both experiments for the traits measured. The analysis was performed using the IPM SPSS statistical package for Windows (version 20.1). Grain yield was used as the dependent variable, and was regressed against all the field- and laboratory-measured traits. The coefficient values estimated were used for the path analysis to determine direct and indirect relationships among the variables measured and analysed for agronomic and quality traits, respectively. Grain yield was used as the independent variable for agronomic and quality traits combined for regression analysis. The path coefficient analysis was estimated as follows [48]:

(11) $$Y=\beta_{0}+\beta_{1}X_{m}+\beta_{2}X+\varepsilon_{1}$$

(12) $$X_{m}=\gamma_{0}+\gamma_{1}X+\varepsilon_{2}$$

where *Y* denotes the dependent variable, *X_m_* is the mediator to the dependent variable, *X* is the exogenous independent variable matrix, $\varepsilon_{1}$ and $\varepsilon_{2}$ are the errors, *β*_0_ and $\gamma_{0}$ are the intercepts, and *β*_1_, *β*_2_, and $\gamma_{1}$ are the regression coefficients to be estimated. The predictable coefficient values *β*_1_, *β*_2_, and $\gamma_{1}$ were used to calculate the impacts of independent variables on dependent variables, where *β*_2_ represents the direct effect of *X* on *Y*, and the magnitude of the indirect effect of *X* on *Y* is estimated by $\gamma_{1}$*β*_1_.

## 5. Conclusions

All the traits studied were highly heritable, with genetic variance dominating environmental variance. The relationship between grain yield and secondary traits, such as ear aspect, ears per plant, and ear rot, indicated that they are important contributors to grain yield. Path analysis identified ears per plant as a medium through which several secondary traits contributed to grain yield indirectly. It is strongly recommended that ear aspect should be considered as a key secondary trait in breeding for QPM hybrids. In addition, due to the negative association between grain yield and tryptophan, and protein and tryptophan, it is recommended that gene pyramiding should be considered for these traits. The full potential of QPM hybrids could be realised if all these traits were incorporated into the genotype.

## Figures and Tables

**Table 1:** Estimates of variance components and heritability for grain yield, and agronomic and quality traits of maize hybrids.

Traits	Variance Components								
	Line Variance	Tester Variance	Line x Tester Variance	Genotype Variance	Additive Variance	Dominance Variance	Environmental Variance	Broad-Sense Heritability (%)	Narrow-Sense Heritability (%)
GY	0.030	0.1800	0.43000	0.5800	2.320000	1.72000	0.13000	0.970	0.560
ASI	0.002	0.0200	0.02400	0.0420	0.167000	0.09700	0.04500	0.854	0.541
EH	56.020	67.9600	24.04000	130.6200	522.460000	96.17000	6.61000	0.990	0.840
EPP	0.001	0.0010	0.00300	0.0050	0.019000	0.01200	0.00100	0.955	0.578
ER	5.320	2.6500	0.74000	7.8700	31.490000	2.96000	7.04000	0.830	0.760
EA	0.004	0.0060	0.01700	0.0250	0.101000	0.06800	0.01000	0.946	0.563
HC	0.117	0.0000	0.02900	0.1470	0.590000	0.11600	0.26500	0.727	0.608
PH	54.170	54.7300	52.73000	147.7000	590.780000	210.92000	9.28000	0.990	0.730
DA	3.720	2.5400	0.68000	6.2900	25.160000	2.73000	0.24000	0.990	0.900
RL	3.060	0.0000	0.00000	2.9000	11.580000	0.00000	7.02000	0.620	0.620
SL	1.210	1.6500	0.46000	2.8700	11.470000	1.83000	4.66000	0.740	0.640
Fibre	0.005	0.0110	0.00500	0.0180	0.074000	0.01900	0.00200	0.978	0.777
Moisture	0.132	0.0590	0.12800	0.2980	1.191000	0.51300	0.23600	0.878	0.614
Oil	0.133	0.0890	0.01400	0.2120	0.849000	0.05600	0.04400	0.954	0.895
Protein	0.133	0.1880	0.06900	0.3440	1.375000	0.27400	0.06300	0.963	0.803
Starch	0.305	0.1950	0.09900	0.5470	2.187000	0.39400	0.15200	0.944	0.800
Tryptophan	0.000	0.0003	0.00001	0.0003	0.001020	0.00003	0.00002	0.983	0.956

GY, grain yield; DA, days-to-anthesis; ASI, anthesis-silking interval; PH, plant height; EH, ear height; EA, ear aspect; ER, ear rot; RL, root lodging; SL, stalk lodging; HC, husk cover; EPP, ear per plant.

**Table 2:** Estimates of principal component analysis of maize hybrids for grain yield and agronomic traits.

Traits	Eigenvectors			
	PC1	PC2	PC3	PC4
Grain yield	0.018	0.012	0.019	−0.021
Plant height	0.718	0.657	−0.211	−0.011
Ear height	0.682	−0.610	0.364	0.003
ASI	0.003	0.005	−0.014	0.019
Days-to-shed	0.095	−0.202	−0.058	−0.067
Root lodging	−0.001	0.111	0.199	0.893
Stalk lodging	0.002	−0.146	−0.191	0.427
Husk cover	−0.006	0.009	0.023	−0.007
Ears per plant	0.001	−0.002	0.000	−0.003
Ear rot	−0.105	0.347	0.861	−0.119
Ear aspect	−0.006	−0.001	0.008	−0.001
Eigenvalue	273.987	23.479	18.174	14.102
Proportion (%)	80.10	6.90	5.30	4.10
Cumulative (%)	80.10	87.00	92.30	96.40

NB: PC, principal component.

**Table 3:** Principal component analysis of maize hybrids for quality traits.

Traits	Eigenvectors			
	PC1	PC2	PC3	PC4
Tryptophan	0.002	−0.003	−0.011	−0.002
Moisture	0.982	−0.065	0.157	0.077
Protein	−0.048	0.811	0.534	0.205
Oil	0.048	0.185	−0.602	0.756
Starch	−0.172	−0.551	0.568	0.557
Fibre	0.030	−0.026	−0.063	−0.266
Eigenvalue	3.562	1.149	0.837	0.029
Proportion (%)	63.663	20.540	14.961	0.521
Cumulative (%)	63.66	84.20	99.16	99.68

NB: PC, principal component.

**Table 4:** Principal component analysis of maize hybrids for grain yield, agronomic and quality traits.

	Eigenvectors					
	PC1	PC2	PC3	PC4	PC5	PC6
Grain yield	0.018	0.013	0.020	−0.020	−0.002	0.215
Plant height	0.718	0.657	−0.211	−0.014	0.068	−0.040
Ear height	0.681	−0.609	0.365	0.007	−0.038	0.069
ASI	0.003	0.005	−0.014	0.019	0.006	0.010
Days-to-anthesis	0.095	−0.201	−0.059	−0.065	0.108	−0.266
Root lodging	−0.001	0.112	0.194	0.892	−0.363	−0.027
Stalk lodging	0.002	−0.144	−0.195	0.429	0.849	−0.041
Husk cover	−0.006	0.010	0.023	−0.007	0.034	0.024
Ears per plant	0.001	−0.002	0.000	−0.003	0.005	0.002
Ear rot	−0.105	0.348	0.859	−0.112	0.317	−0.096
Ear aspect	−0.006	−0.001	0.008	−0.001	−0.002	−0.034
Tryptophan	0.000	0.001	−0.001	0.001	0.001	0.000
Moisture	−0.010	0.043	0.031	0.014	0.107	0.912
Protein	0.007	−0.010	0.046	−0.017	−0.128	0.045
Oil	0.006	0.010	−0.026	0.030	0.010	0.022
Starch	−0.007	−0.014	0.021	−0.024	0.016	−0.171
Fibre	−0.002	0.009	−0.004	0.003	0.006	0.019
Eigenvalue	274.053	23.527	18.247	14.127	8.378	3.628
Proportion (%)	78.80	6.800	5.30	4.10	2.40	1.00
Cumulative (%)	78.80	85.60	90.80	94.90	97.30	98.40

NB: PC, principal component.

**Table 5:** Phenotypic correlation (r_P_) (above diagonal) and genotypic correlation (r_G_) coefficients (below diagonal) between grain yield, and agronomic and quality traits of maize hybrids.

Traits	GY	DA	ASI	PH	EH	RL	SL	EPP	HC	ER	EA	Trpt	Mois	Prot	Oil	Fibre	Starch
GY	-	−0.16	−0.14	0.29 **	0.19 *	0.18 *	−0.19 *	0.42 **	−0.1	0.06	−0.54 **	−0.27 **	0.26 **	0.25 *	0.07	−0.06	−0.17
DA	−0.19 **	-	0.35 **	0.39 **	0.60 **	0.02	0.22 *	−0.04	−0.33 **	−0.29 **	0.01	−0.10	0.03	−0.07	0.13	−0.27 **	0.02
ASI	−0.23 **	0.78 **	-	0.09	0.14	0.15	0.32 **	−0.29 **	0.08	−0.09	0.14	0.10	0.19*	−0.24 *	0.14	0.18 *	−0.01
PH	0.40 **	0.43 **	0.30 **	-	0.82 **	0.20 *	0.14	0.15	−0.15	−0.17	−0.53 **	−0.09	0.01	0.08	0.14	−0.07	−0.09
EH	0.27 **	0.65 **	0.37**	0.86 **	-	0.20 *	0.19 *	0.14	−0.23 *	−0.24 *	−0.44 **	−0.23 *	−0.07	0.18 *	0.04	−0.26 **	−0.01
RL	NA	NA	NA	NA	NA	-	0.13	−0.03	0.23 *	−0.03	−0.18 *	−0.18 *	0.01	0.14	0.14	−0.07	0.04
SL	−0.28 **	0.47 **	1.00 **	0.37 **	0.43 **	NA	-	−0.08	0.03	−0.20 **	−0.13	0.19 *	0.04	−0.26 **	−0.02	−0.09	0.21 *
EPP	0.64 **	−0.08	−0.76 **	0.19 *	0.20 *	NA	−0.16	-	−0.20 *	0.05	−0.34 **	−0.10	0.01	0.05	−0.04	−0.17	0.07
HC	−0.16	−0.68 **	0.21 *	−0.24 *	−0.41 **	NA	0.06	−0.63 **	-	0.18 *	0.06	0.11	−0.05	−0.01	−0.10	0.16	0.06
ER	0.29 **	−0.47 **	−0.35 **	−0.20 *	−0.39 **	NA	−0.88 **	0.31 **	0.36 **	-	0.28 **	−0.03	0.12	0.06	−0.03	0.16	−0.09
EA	−0.83 **	0.01	0.27 **	−0.83 **	−0.74 **	NA	−0.68 **	−0.58 *	−0.02	0.11	-	0.19*	−0.02	−0.14	0.05	0.14	−0.08
Trpt	−0.39 **	−0.12	0.16	−0.12	−0.26 **	NA	0.42 **	−0.18 *	0.24 *	−0.04	0.35 **	-	0.15	−0.56 **	0.51 **	0.39 **	−0.30 **
Mois	0.50 **	0.05	0.38 **	0.02	−0.10	NA	0.05	0.06	−0.06	0.15	−0.07	0.21 *	-	−0.32 **	0.20 *	0.48 **	−0.20 *
Prot	0.38 **	−0.04	−0.45 **	0.11	0.23 *	NA	−0.54 **	0.15	−0.01	0.07	−0.31 **	−0.64 **	−0.45 **	-	−0.15	−0.40 **	−0.30 **
Oil	0.04	0.14	0.21 *	0.14	0.03	NA	−0.16	−0.09	−0.23 *	−0.05	0.13	0.58 **	0.32 **	−0.17 *	-	0.29 **	−0.69 **
Fibre	−0.10	−0.31 **	0.28 **	−0.09	−0.31 **	NA	−0.19 *	−0.26 **	0.31 **	0.24 **	0.20 *	0.44 **	0.64 **	−0.48 **	0.33 **	-	−0.32 **
Starch	−0.25*	0.01	0.01	−0.12	−0.02	NA	0.44 **	0.04	0.16	−0.13	−0.08	−0.35 **	−0.29 **	−0.21 *	−0.78 **	−0.36 **	-

GY, grain yield; DA, days-to-anthesis; ASI, anthesis-silking interval; PH, plant height; EH, ear height; EA, ear aspect; RL, root lodging; SL, stalk lodging; HC, husk cover; ER, ear rot; EPP, ear per plant; Trypt, tryptophan; Mois, moisture; Prot, protein. * *p* < 0.05, ** *p* < 0.01; NA, not applicable.

## Data Availability

The datasets generated during the present study are available from the lead and corresponding authors on request due to privacy restrictions.

## Supplementary Materials

The following supporting information can be downloaded at: https://www.mdpi.com/article/10.3390/plants11060713/s1, Table S1: Site, weather and soil descriptions for the test locations in South Africa and Zimbabwe.
