# Supplementary material for: Heritability and Associations among Grain Yield and Quality Traits in Quality Protein Maize (QPM) and Non-QPM Hybrids
Source: Plants (Basel). 2022 Mar 8;11(6):713. doi: 10.3390/plants11060713 (PMC8951685; doi:10.3390/plants11060713)
Supplement: Supplementary file 1 [file plants-11-00713-s001.zip › plants-1585219-supplementary.pdf]

Supplementary Table S1. Site, weather and soil descriptions for the test locations in South Africa and Zimbabwe

Location name	Country	Latitude	Longitude	Elevation above sea level (m)	Total rainfall (mm)	Mean Temperature ranges (°C)		soil parameter			
						Minimum	Maximum	N (g kg^{-1})	P (mg kg^{-1})	K (mg kg^{-1})	Soil pH
Harare	Zimbabwe	-17°46'S	31°02'E	1406	820	5..5	23.8	0.5	18.13	0.6	5.8
Gwebi	Zimbabwe	-17°13'S	31°E'	1406	637	4.9	26.2	0.4	14.5	1.3	6
Bindura	Zimbabwe	-17 °18′S	31° 02'E	1480	976	4.8	27.8	0.3	19.3	2.4	6
Harare-misting	Zimbabwe	-17°46'S	31°02'E	1406	820	5.5	23.8	0.8	22.36	1.3	5.8
Rattrey-Arnold	Zimbabwe	-17° 67′S	31° 17′E	1462	865	5.3	23.6	0.7	17.2	3.1	6
Glandel	Zimbabwe	-17°31'S	31°3'E	1147	669	7.4	28.1	0.5	11.4	2.2	6
Lionsdel	Zimbabwe	-17°25′S	30° 02′E	1232	620	6.1	29	0.4	15.7	1.7	6
Cedara 2018	South Africa	-29°54'S	30°26'E	1066	807	9.4	27.1	0.7	11.3	77.9	5.6
PotchTB8-18	South Africa	-26°73'S	27°08'E	1349	544	13.4	28.6	0.3	27.2	278.4	6.4
PotchR8-18	South Africa	-26°73'S	27°06'E	1349	544	13.4	28.6	0.5	16.6	278.4	6.1
Potch 2019	South Africa	-26°74'S	27°08'E	1349	539	11.6	27.8	0.4	27.1	280.6	6.4
Cedara 2019	South Africa	-29°71'S	30°26'E	1066	559	8.5	25.9	0.6	10.9	81.3	5.9
